# What We Mean By ‘Values in Healthcare’: The Importance of Reaching a Consensus

**DOI:** 10.7759/cureus.79034

**Published:** 2025-02-15

**Authors:** Joachim P Sturmberg, Saadi Taher

**Affiliations:** 1 General Practice, University of Newcastle, Newcastle, AUS; 2 Research, Central Coast Research Institute, Gosford, AUS; 3 Nephrology, Health System Transformation Program, Ministry of Health, Riyadh, SAU

**Keywords:** complexity science, complexity thinking, ethics and professionalism, healthcare financing, health policy and economics, health system policy, health system redesign, philosophy of medicine, value-based medicine, values

## Abstract

The concept of ‘values’ in healthcare is widely debated, with no universal definition despite its central role in health system reform. This paper does not seek to define value narrowly but instead aims to stimulate a generative discussion among stakeholders. Using a systems-thinking framework, we explore four key perspectives: the subjective and value-laden nature of ‘value,’ the influence of financial interests, the role of personal values in shaping care delivery, and the potential for a shared human-centred value framework. By highlighting the diverse and often conflicting interpretations of value, we encourage an inclusive dialogue to guide the development of health systems that are equitable, patient-centred, and sustainable, benefiting individual stakeholders and society at large.

## Editorial

Values in healthcare: the importance of reaching a consensus

Values in healthcare has re-emerged as the new buzzword amongst those involved in health system reform. However, the concept of value in healthcare is not a new one. In 1908, William Mayo addressed it this way: “The best interest of the patient is the only interest to be considered, and in order that the sick may have the benefit of advancing knowledge, union of forces is necessary” [1]. He suggested that the value of healthcare arises from a shared understanding and collaborative effort of everyone involved in providing care.

The term ‘value-based healthcare’ lacks a universally accepted definition, and there is no consensus on what ‘value’ in health truly means [2,3]. In this essay, we aim to stimulate a conversation amongst all stakeholders within the health care system to consider the concept of ‘value’ from four distinct perspectives. First, we highlight the inherent complexities of the term ‘value’ as itself being ‘problematically value-laden’ with subjective judgements and biases. This allows stakeholders to exploit the term to further their pecuniary interests at the expense of collaborative efforts to advance the health system as-a-whole to deliver those outcomes that meet its patients’ needs. We then explore how the pecuniary interest perspective has shaped the ‘value-based healthcare’ debate (largely in the US). We examine the underlying complex drivers and analyse their far-reaching implications for the organisation and delivery of health care. The concluding section illustrates how embracing a shared ‘human-focused value frame’ can yield both, high-quality patient-focused care outcomes as well as sustainable business practices.

Our essay has three aims, one, to elaborate the systemic nature of the health system, two, to highlight the interdependencies between stakeholder appreciations that shape the various frames of ‘values in health care/healthcare systems’, and lastly, to emphasise that a seamlessly integrated health system relies on a common understanding of purpose based on shared values.

Part 1: A philosophical discourse

The Multiple Meanings of ‘Value’

People rightly contest the notion of ‘value’ since there are many different definitions clustering around two broad frames - business and humanism (Table 1). In business and economics, 'value' refers to efficiencies, invariably relating to concepts of return on investment and profit. The humanistic perspective can be divided into an ethical domain of personal reflection - in particular intrinsic attributes of autonomy, authenticity, benevolence, respect, and resilience, and a social domain of collaborative effectiveness - focusing on collaboration, commitment, equity, resilience, respect, and sustainability [4-9].

**Table 1:** The Values Associated with a Business and a Humanistic Values Frame

Domains	Associated values
Values in the Business frame (focus on efficiency)	
Business	Return on Investment (ROI)
	Profit
Economics	Products
	Services
	Customers
	Employers
	Markets
		Values in the Humanistic frame	
Ethical domain	
Personal Appreciation	Intrinsic Values (autonomy, authenticity, benevolence, resilience, respect)
	Accountability
	Equity
	Wisdom
Effectiveness	Societal & Social Groups (collaboration, commitment, equity, resilience, respect, sustainability)
	Culture and Meaning
	Religion

Mental Frames

Mental frames ‘define’ our various worldviews, which in turn determine our ‘value’ preferences, like a focus on money, power and control, mindfulness, or social responsibilities [10]. They define the boundaries within which we predominantly think about an issue, thus affecting how we approach and envision solutions to problems. They must be ‘frame coherent’ to avoid cognitive dissonance, and thus discomfort or distress [10].

Of equal importance is the distinction between ‘health care’ and ‘healthcare’ (though often used synonymously). Health care refers to the activities involved in attending to patients’ complaints and needs, whereas healthcare (as in healthcare system) refers to the various structural domains (policy, physical infrastructure, health professionals) required to care for individuals and populations [11].

While these frames reflect the distinct prerogatives of individual stakeholder, the health system as-a-whole encompasses all of these perspectives. For the system to function in a seamlessly integrated way - a prerequisite to delivering patients’ desired health outcomes - the system requires a mutually agreed-upon value proposition, to ensure the system operates effectively, efficiently and equitably for the benefit of society as-a-whole [12,13].

Our Supposition

Health care produces a ‘common good’ benefit for society, with its value surpassing the sum of either individual financial or personal rewards. Fundamentally, based on first principles, the inherent values of health care are interconnected and thus inseparable, i.e. they are, by definition, systemic, and neither good or bad, or right or wrong.

How to achieve a ‘common good’ benefit for society remains an open question. We have no particular preference for how healthcare is organised in terms of private, not-for-profit or public organisational structures, provided the chosen arrangements achieve the desired ‘common good’ outcome.

A Systemic Understanding of the ‘Healthcare System’

Capra highlighted a fundamental distinction in approaches to problem-solving, contrasting a reductive versus systemic frame. The reductive or self-assertive approach involves rational, analytic, linear and reductionist thinking, emphasising expansion, competition, domination and quantitative values. In contrast, the systemic or integrative approach involves intuitive, synthesised, nonlinear and holistic thinking, focusing on conservation, cooperation, partnership and qualitative values [14]. To avoid any conceptual confusion we will first briefly clarify the ‘systems science’ definitions of systems and their key characteristics (a more detailed discussion can be found here [13]). 

The Defining Characteristics of a System

A system is defined as-a-whole that cannot be divided into independent parts. In a system the parts are interconnected and interdependent, which in turn means that the behaviour of any part depends on the behaviour of the other parts; no part has an independent behaviour. Since the parts are interconnected and interdependent there are multiple pathways that explain the system’s emergent dynamics [15].

Furthermore, every system is always simultaneously part of a larger supra-system while being itself composed of various sub-systems. The function of a system requires that every part, despite its unique ‘part’s purpose’, must align its behaviour to support the system as-a-whole.

The most important feature of a system is that the properties of the whole are not present in its parts. Understanding a system part (through the traditional reductionist approaches) does not provide insights into how the system functions as-a-whole, and how it functions in its particular context. And lastly, systems whose behaviours are altered by their interdependent dynamics have emergent behaviours, and are referred to as complex adaptive systems (CAS) [13].

Visualising a Complex Adaptive System

**Figure 1:**
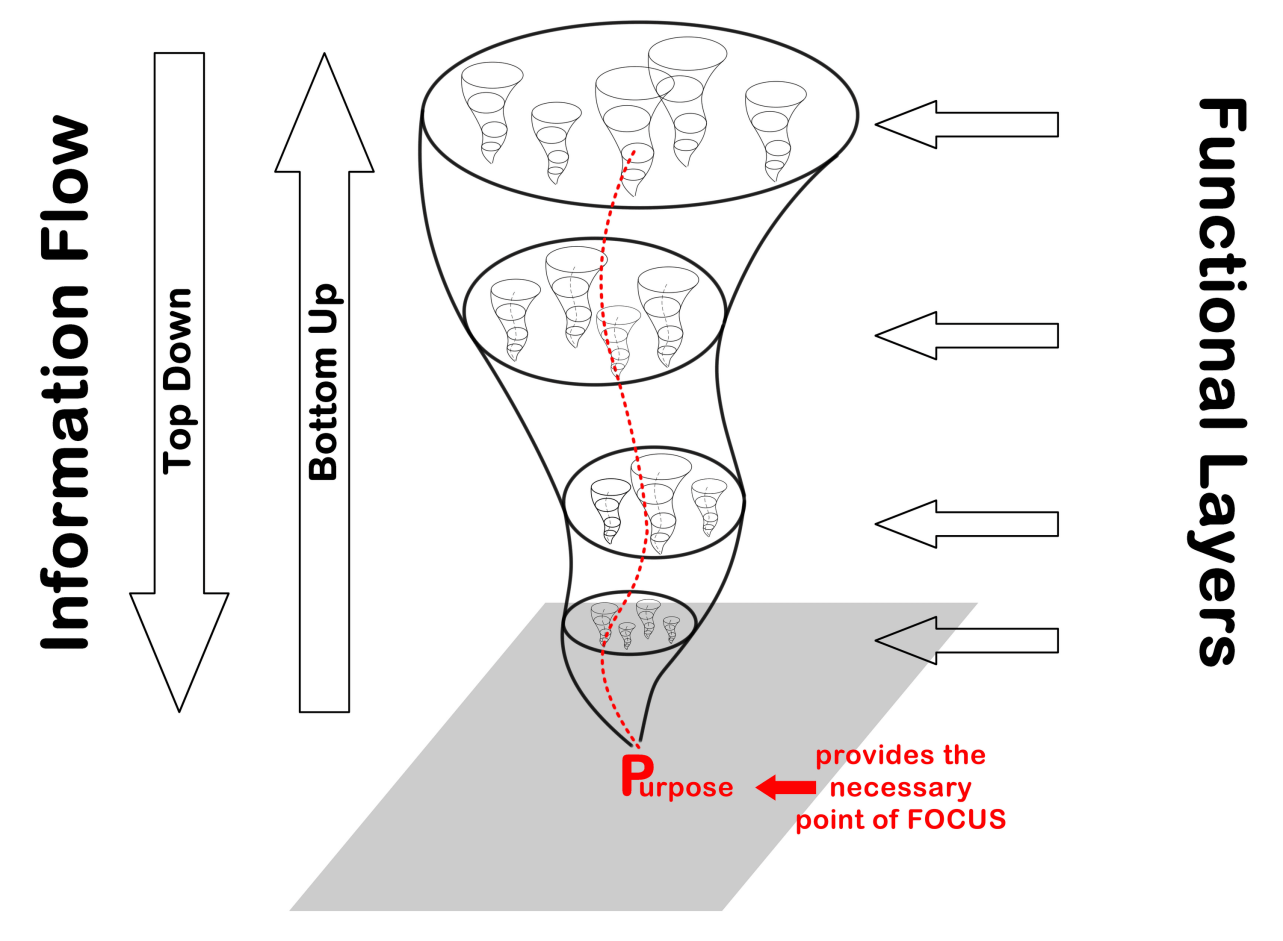
The Nested Nature of a System Emerges from the Focus on its PURPOSE The key structural and dynamic characteristics of an organisational system are: it is functionally layered to carry out the diverse activities to enable the realisation of its overall purpose; it is simultaneously constituted of subsystems while itself being part of a larger suprasystem; its dynamic behaviours depend on top-down information/resource flows that constraint downstream activities, but depend on the bottom-up feedback to fine tune those in light of emergent changes. (Author created image)

The nature and dynamics of a CAS are much easier to appreciate visually, such as through the metaphor of a vortex (Figure 1). The vortex metaphor illustrates a system’s key properties - for a vortex to emerge, it requires a focal point (= purpose), it has a flexible structure (= the interconnectedness between its components) that is maintained by its bottom-up, top-down information flows (= the interdependency of responses in light of what other components are doing). These attributes make a vortex inherently robust to perturbations. As long as the system maintains its focal point (= adheres to its purpose) it will re-stabilise itself following a perturbation, returning close to its original state [13].

Organisations are Socially Constructed Functionally Layered Systems

The health system, like all other systems involving human engagement, is a socially constructed organisational system [16]. According to Morin, organisation [as a process] “binds elements in relationships that thus become components of a whole … with qualities unknown to these components outside the structure”, thus the ‘connecting of’ the parts that constitute an organisation [as a structure] gives rise to its complexity [17]. The driving force of organisation [the process] arises from achieving a desired purpose - in this case to improve people’s health. This requires a collaborative way of working together across its organisational scales and to remain focused on what needs to be achieved. Put differently, the organisation has to seamlessly work as-a-whole within and across its organisational layers to realise its purpose [13].

The complex adaptive nature of an organisation emerges as it evolves. Organisations typically begin as a ‘one-man show’, where a single individual starts an enterprise and soon requires some help. Initially, new members clearly understand the organisation’s purpose and easily work together to achieve its goals. As the organisation grows, it inevitably has to create functional units to manage specific domains, resulting in a functional layering of the organisation. These functional layers, by some form of necessity, typically define their own purposes which cause them to lose sight of the organisation overall purpose. This in turn leads to a mismatch of information flows and the emergence of dysfunctional behaviours - the people doing the frontline work no longer have the necessary unequivocal understanding of 'how to do what' [12].

All Organisational Systems Are Perfectly Designed to Get the Results They Get

It may sound like a cliche; however, systems sciences have shown us how the structure (interconnectedness) and function (interdependencies) of a system achieve the outcomes they are designed for. Another crucial factor to system function is context - achieving desired outcomes will necessarily vary in light of environmental constraints [18,19].

Fundamentally then, organisational failure stems from a lack of appreciation for and utilisation of the interdependence and variable pathways between an organisation’s parts resulting in dysfunctional dynamics. These dysfunctions frequently arise from ‘improvement efforts’ focused on improving a ‘part’ without giving due consideration to its effects on the rest of the system [16].

Part 2: Values and the health system

In the literature, ‘increasing value’ is often equated with quality improvement. However, we would argue, this is a misunderstanding, as one’s values determine the type of quality one aims to achieve. These differences are evident in the value discussions between Berwick’s and Porter’s perspectives.

The ‘Value Movement’

As far back as 1992, Berwick articulated that the value of health care arrives from a systemic organisation of care [20]. He emphasised that understanding the purpose of health care is paramount - without a purpose, there is no system. Berwick clearly sees the purpose of health care being to preserve, restore and improve the health of our patients [21]. A systemic perspective entails that health care extends beyond the narrow boundaries of medical care to include a whole range of external services, ranging from pre-schooling to social care and the work environment. Berwick puts the patient’s needs at the centre of care and emphasises the role of health care leadership as facilitating service delivery that encompasses all care needs [20].

Achieving a values-based re-design of the (American) health system is challenging, fundamentally arising from the business and profit value understanding ingrained in her culture. Berwick promotes a number of strategies that might shift the values that define the purpose of a re-designed health system. This requires ‘transformative leadership’ that amongst other things enables the creation of a community voice that articulates healthcare needs in a way they are experienced, expands our knowledge to match our purpose, starts a community-based healthcare planning process, aligns financing with the system’s purpose, and pushes for an increase in investments in learning across the board [20]. 

Porter defines value as the achievement of ‘outcomes that matter to patients’ relative to costs. To achieve this he proposes six steps: organising around patients' medical condition rather than physicians' medical specialty, measuring costs and outcomes for each patient, developing bundled prices for the full care cycle, integrating care across separate facilities, expanding geographic reach, and building an enabling IT platform [22]. These changes undoubtedly are part of achieving value, however, they involve as much changes of individual attitudes as structural change to working styles and workplace infrastructure. Although necessary for improving the ‘instrumental component of care’, they are by no means a guarantee to achieve the ‘outcomes that matter to patients’ - that requires to a priori define what ‘matters to this patient’ under my care [23].

Porter further defines patient value as arising from the experience of the cycle of care - like health status achieved; time, complications, and suffering involved in care; and sustainability of benefits achieved; and suggest to measure value in terms of functional status achieved [24]. While functional health status can be measured in a standardised way, this by no means equates to the ‘outcomes that matters to this patient’. Outcomes that matter are highly context sensitive, and what may under one condition be a bad outcome can be an excellent one under a different condition.

The value movement has created a - in our view rather simplistic - formula to determine value:

$\textbf{V} (\text{Value}) = \frac{\textbf{Q} (\text{Quality}) + \textbf{S} (\text{Service})}{\text{Cost} (\textbf{\$})}$

The formula looks neat, but it assumes that its variables can be reliably defined and measured, and therefore treated as ‘value and context free’. However, as the health economist Reinhardt highlighted, quality (or outcomes in healthcare) typically has multiple dimensions and thus cannot be collapsed into a single variable. An even more fundamental problem is the relationship between cost and value; as Reinhardt noted, “the value of a ‘thing’ has nothing whatsoever to do with the cost of its production” [25,26].

Organisations like the OECD approach healthcare from an economic values perspective, focusing on questions such as how much it costs to provide care and who should bear those costs. Cost-containment thinking leads to the bureaucratic solutions like institutional performance management, diagnostic-related group payment systems, and cost-effectiveness analysis of services [27] that stand in the way of ‘delivering what matters’. Furthermore, economic theory considers resource allocation not only as an expenditure/costs but also as an investment, a notion strongly supported by leading medical and economic thinkers [28,29]. These investments lead to broader societal benefits [30,31] and thus benefit the overall productivity and well-being of communities/countries, a point lost in the narrow economo-centric cost-benefit narrative. Growing evidence suggests that the excessive focus on ‘narrow’ economic values can lead to reduced quality of care and poorer patient outcomes [32,33]. 

The European Commission Expert Panel on Effective Ways of Investing in Health has adopted a very different approach to defining value in healthcare, rooted in the solidarity principle. They view value as being both a value as such as a structuring principle for health care delivery and organisation. Their broader definition frames “value-based healthcare (VBHC) as a comprehensive concept built on four value-pillars: appropriate care to achieve patients’ personal goals (personal value), achievement of best possible outcomes with available resources (technical value), equitable resource distribution across all patient groups (allocative value) and contribution of healthcare to social participation and connectedness (societal value)” [2]. These perspectives closely align with those expressed by the National Academies of Sciences, Engineering and Medicine’s report on ‘Crossing the Global Quality Chasm: Improving Health Care Worldwide’ [34].

Value of the Healthcare System

Organisational systems arise from a well-defined and well-understood purpose. To work as a seamlessly integrated system, they require a set of shared values, or core values. Purpose and values are firmly linked and thus must - a priori - be considered and agreed upon by all its members [13].

For an organisation, core values are fundamental. They remain unchanged in a changing environment and form the basis for solving emerging problems and conflicts. Solutions to a problem that are better than a band aid must align as much with the system’s purpose as core values. Only then will they improve everyone’s function, i.e. improve the organisation as-a-whole [16].

Arriving at Core Values

Defining core values is by no means straightforward. As Collins and Porras [35] have alluded to, core values have a personal as well as a group dimension. We must acknowledge that as individuals we have a set of values that frame our worldview, and thereby ‘determine’ how we conduct ourselves in life and work [10].

For an organisation to become a lasting entity, Collins and Porras suggest that the organisation as-a-whole contemplates three questions to define its core values:

One, will they still be valid in a hundred years’ time; two, would you still hold onto them even if they stand in the way of competitive advantage; and three, what are values you would build into an organisation regardless of a specific industry?

Core Values in a Multi-Stakeholder Health System

The health system is a multi-stakeholder system where every part contributes to the whole in a unique way. However, every stakeholder, being a system in their own right, also has an innate perspective on what they regard as their purpose and therefore what they value.

To achieve a well-functioning complex adaptive health system, all its stakeholders must ultimately agree on the system's purpose and values, or as Deming [36] stated: Without an aim, there is no system. This requires visionary leadership to facilitate this process with an unapologetic commitment to remind stakeholders about the interdependent effects of their views. While it is important to acknowledge that business, economic, and social and ethical perspectives coexist within the health system it is equally important to emphasise that only a common perspective shapes the health system’s priorities like access to healthcare, income streams and profits, care environments, care delivery, population health, resource allocation, and equity and justice.

**Figure 2:**
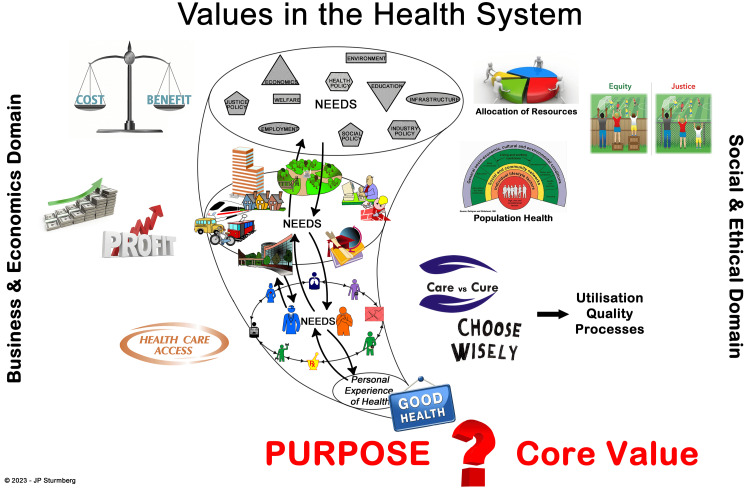
The Stakeholder Diversity of Values in the Health System Note: In a purpose-focused system the various stakeholders’ value prerogatives will align to an agreed core values set. (Author created image)

Figure 2 illustrates the functional layering of the health system within the vortex frame. It clearly conveys to all stakeholders that they are ‘only’ subsystems of the health system as-a-whole and that they must adapt their own purpose and values to align with the health system’s overarching purpose and core values.

The Effect of Values on the Health System 

**Figure 3:**
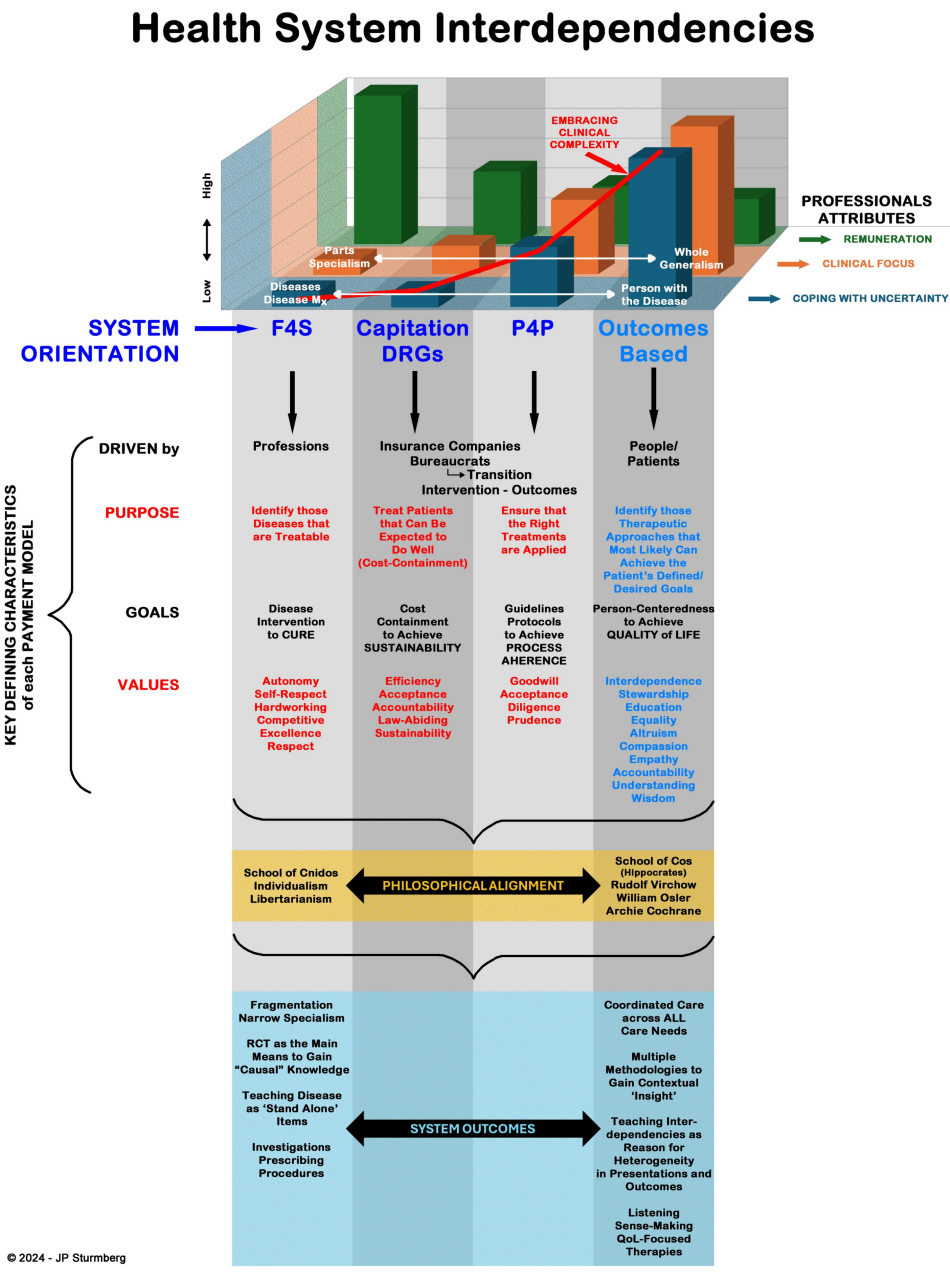
The Complex Interactions between Professional Attributes, System Orientation and Organisational Behaviours Rudolf Virchow – "Medicine is a social science and politics is nothing else but medicine on a large scale. Medicine as a social science, as the science of human beings, has the obligation to point out problems and to attempt their theoretical solution; the politician, the practical anthropologist, must find the means for their actual solution" [39]. William Osler – "Listen to your patient; he is telling you the diagnosis. or It is much more important to know what sort of a patient has a disease than what sort of a disease a patient has" [40]. Archie Cochrane – "In particular, the need for care is widespread while the need for care is widespread, and that the pursuit of cure at all costs may restrict the supply of care" [41]. (Author created image)

As organisations are, strictly speaking, complex adaptive systems [16,37,38], we cannot determine a-priori which of the multiple domains, or which specific factor, shape a health system. However, by analysing a health system in a backward fashion - starting with the outcomes it produces - we can reveal key interdependencies and their consequences, and thereby identify a system’s underlying value attributes (Figure 3).

We identified four health system orientations. It is important to emphasise that the underlying attributes of each orientation occur on a continuum and should not inadvertently be seen as either privileged or deterministic. Nevertheless, it should become obvious that the degree of orientation impacts the interactions with other attributes, and that these interactions ultimately lead to the system’s achievements. Contemporary discourses focus on the financing and financial stability of the health system; hence, we use this as the starting point for our analysis.

Payment Systems and Professional Attributes

Different payment systems align with particular professional attributes, especially in terms of how professionals cope with uncertainty, their clinical focus, and the importance they place on remuneration.

(a) Fee-for-service (F4S) models, for example, tend to attract professionals who prefer a narrower focus on specific disciplines. This approach minimises uncertainty and often guarantees higher incomes [42-45].

(b) Outcomes-Based Payments and Generalist Practice - In contrast, outcomes-based payment models are better suited to professionals who are comfortable managing the inherent uncertainties of undifferentiated practice, such as generalists. These professionals typically place less emphasis on remuneration and more on broader patient-centred outcomes [43,46].

(c) Complexity and Cognitive Workload - An often-overlooked factor is the relationship between professional attributes and the complexity of clinical work. Specialists, with their narrow disease-focused practice, face significantly less cognitive workload compared to generalists, who manage a broader range of illness presentations and whole-person care [46-48].

(d) Impact on Burnout - This difference in cognitive workload can have profound implications for professional well-being. The increased complexity associated with generalist practice raises the risk of professional burnout, a challenge that requires systemic attention [47,49].

Health System Orientation and Financial Focus

Health systems are often oriented by their financial models, which include fee-for-service (F4S), capitation or payment via diagnostic-related groups (DRGs), pay-for-performance (P4P), and outcomes-based payments. Each of these models shapes how care is organised, delivered, and evaluated [44].

Consequences of Financial Models

Adopting any of these payment systems has both intended and unintended consequences. For example, while fee-for-service incentivises service volume, it may lead to fragmented care. Similarly, outcomes-based payments encourage holistic care but can place additional cognitive and administrative burdens on healthcare providers [43].

(a) Organisational perspectives: Purpose, goals, values and ‘simple (or operational)’ rules determine the behaviours and outcomes of a system. Each of these payment systems is preferred by vested interests, and drives the system, whether it be professionals, insurance companies/bureaucrats, or patients [44].

(b) Purpose of fee-for-service systems: The F4S model primarily focuses on identifying diseases and preferentially treating those that can be cured. This approach incentivises volume but may overlook the broader aspects of patient care.

(c) Capitation and cost containment: In comparison, capitation and DRG, along with P4P payment systems, emphasise cost containment. These models often prioritise treating patients who are more likely to benefit from care or adhering to pre-defined management schedules, which may restrict flexibility in care delivery.

(d) Outcomes-oriented Models: Outcomes-oriented payment systems take a different approach by emphasising the importance of understanding patients’ illnesses. These systems encourage collaborative efforts to achieve person-defined quality-of-life outcomes, aligning care delivery with individual needs and values.

(e) Values underlying payment systems: The values underlying the F4S, capitation/DRG and P4P orientation hence differ notably. The former consists of predominantly business/economics value sets and preferentially attracts professionals whose personal values strongly align with autonomy, excellence, and competitiveness, whereas the latter entails mostly those from the ethical and social set and preferentially attracts professionals whose personal values strongly align with altruism, equity and justice. 

Key differences between payment models and their impacts are summarised in Table 2.

**Table 2:** Comparisons of Payment Methods FFS - Fee-For-Service; DRGs - Diagnostic Related Groups; P4P - Pay for Performance. (Compiled by authors from various sources [50-53])

Payment Model	Payment Structure	Incentives	Pros	Cons
FFS	Per service	Quantity	Access, choice	Overutilisation, high costs
Capitation	Fixed per patient	Efficiency	Cost control, simplicity	Underutilisation risk
DRGs	Fixed per diagnosis	Efficiency	Cost control, hospital efficiency	Complexity variability
P4P	Performance-based	Quality	Quality improvement	Complexity, gaming risk
Outcomes-Based	Outcome-based	Quality & Efficiency	Focus on outcomes, efficiency	Measurement challenges

Philosophical Perspectives

The focus on disease or personal illness has ancient roots, dating back to the Greek Schools of Cnidus (disease-focus) and Cos (illness-focus). Modern perspectives relate the disease focus with the philosophical notions of individualism and libertarianism, while the personal illness focus aligns with humanitarian notions of social equity (Virchow), understanding the patient (Osler), and humility (Cochrane). Each perspective is underpinned by a unique set of values. 

Part 3: Mind frames, values and system impacts

Organisational systems are designed, and their design is - more often subtly than overtly - predicated on the designer’s mind frame [10], which in turn shapes the orientation of the system’s purpose. As a mind frame is closely linked to a particular value set [10], it might be difficult to determine if values define the system orientation, or if the system orientation determines its values - ultimately, it is a circular argument. Regardless of the sequence, both are interdependent and shape the system’s dynamics, which in turn allows the emergence of its (build-in) outcome patterns. These patterns include system purpose, access and equity to the health system, health system organisation, the focus of care provision, carer pathways, individual and population health outcomes, selection of new graduates, teaching, research, and so forth.

Implications of a Business Value Frame

The business framework is built on the principles of the free market, individualism, and choice. It operates most effectively when targeting a well-defined market segment, naturally fostering a mindset of ‘market segmentation.’ However, this focus often comes at the expense of comprehensive, whole-person care, instead favouring narrowly focused specialised, single-disease approaches. Managing individual diseases aligns more readily with structured business strategies centred on efficiency and control, leading to the widespread adoption of guidelines and protocols [54]. As a corollary, it tends to avoid an engagement with patients’ needs to address the complexities and uncertainties that patients inherently face in their conditions [47].

The reasoning within this framework is shaped - and constrained - by factual evidence derived primarily from randomised controlled trials, which are upheld as the ‘gold standard’ of knowledge creation [55,56]. This perspective has unduly influenced medical education, emphasising disease-specific knowledge, and clinical practice that prioritises investigations, pharmacotherapy and technical procedures [57-60]. Furthermore, the business framework necessitates an organisational management structure anchored in ‘command and control’ principles, further reinforcing its structured, efficiency-driven approach.

Implications of a Humanistic Value Frame

The humanistic frame ‘values’ a whole-person orientation that aims to understand the patient’s illness and to identify all his care needs across the somatic, social, emotional, and cognitive domains [34,61,62]. It embraces multiple research methodologies to gain contextual insights about the person’s illness experiences and trajectory [63]. Medical teaching emphasises that interdependencies are the reason for the heterogeneity in patient presentations, treatment choices and health outcomes [58,60,64]. A humanistic values frame shapes a consultation style that prioritises listening and sense-making, and treatment approaches focused on shared decision-making to achieve the patient’s best possible quality of life outcomes [47,65,66]. Organisational leadership accepts the need for flexible workplace and delivery approaches, and the need to empower and support its members to adapt in light of changing needs and circumstances [67]. 

The Implications of a Purpose and Value Set on System Function 

**Figure 4:**
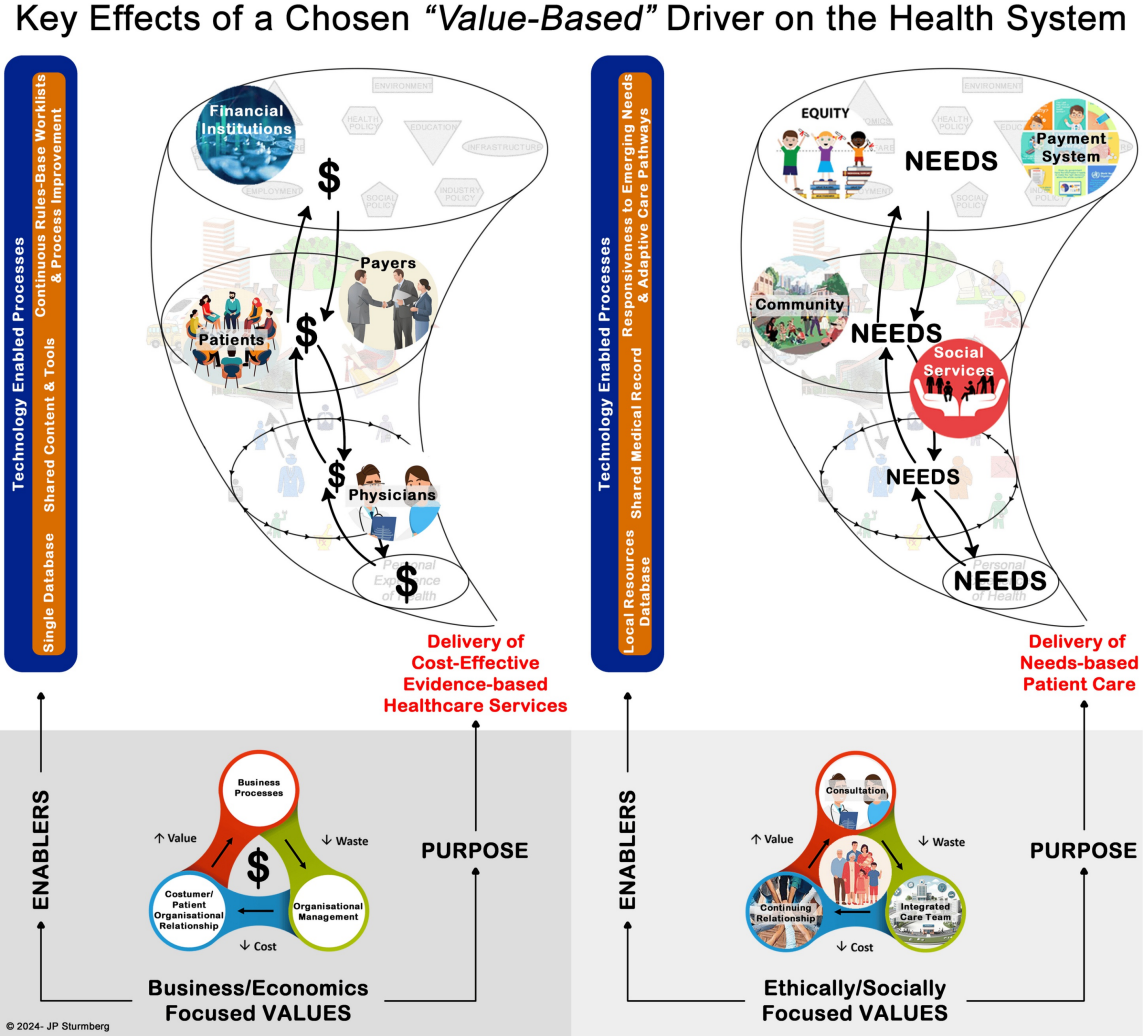
Implications of Value Propositions on the Emergence of the Health Note that the principal idea – reducing waste and cost to increase value – is achievable with both approaches, however they reflect very different *value propositions* and result in different *interactions *and *outcomes*. The centre of the image shows the driver and the resulting activities leading to waste and cost reduction. Key effects on the functional levels are shown in the vortex representation. (Author created image)

Figure 4 visualises how business and humanistic mind frames shape the drivers, and thereby the health system’s organisation and dynamics. The business perspective, focused on processes, organisational management and organisational relationships, stands in stark contrast to the humanistic perspective, which is focused on consultation, integration of care and continuing relationships. Understanding these two mind frames and their inherent value sets is a prerequisite for achieving the common understanding needed to define potential pathways towards achieving an effective, efficient, and equitable health system arising from shared values.

To clarify, neither frame should be regarded as either right or wrong nor better or worse. Rather, it is important to understand that each frame entails ‘build-in’ system-wide consequences that require deliberation before agreeing and implementing a particular change.

Part 4: Achieving lasting value-based improvement

The discourse so far has highlighted the different - highly interconnected and interdependent - dimensions that contribute to a ‘values-based health system’. The underlying attributes as well as their implications on shaping a health system should not be seen as dichotomous; they exist on a continuum and rightly reflect the necessary diversity of perspectives to achieve the common goal.

Values matter greatly as they determine ‘what matters’ and ‘how what matters gets realised’ [3,12,13]. We have also alluded to the intricate web that shapes one’s values, and how they are embedded within health system orientations and outcomes. Lastly, we have indicated the way in which values are the basis from which a coherent system emerges.

Re-Building a Values-Based Health System: A Systemic Approach

Key to achieving a values-based health system is the embracement of systemic thinking, especially the realisation that in a complex adaptive system, everything is connected to everything else, and changes to one part change all other parts.

This in particular means that we need to appreciate the contextual nature of a health system. Many health problems arise, as Virchow [39] already emphasised, from our patients’ social and ecological environments. What he did not know at the time is how experiencing injustices, discrimination of age, gender and culture, physical and/or emotional abuse, the promotion of unhealthy lifestyles, and exposure to destructive and/or toxic environmental conditions activate the biological pathways that lead to premature morbidity and mortality [68-71].

The re-design of a values-based health system must start with embracing the complex adaptive nature of the various system dimensions, above all, health itself, health service organisation, health system orientation, and leadership. Put differently, systems thinking must be embraced as the sine-qua-non to realise ‘value’, i.e. safe, effective, person-centred, accessible, timely, affordable, efficient and equitable health care [13,34].

The Systemic Understanding of Health 

The WHO definition of health has been criticised for being overly idealistic and failing to account for the dynamic nature of health, especially the roles of adaptability, resilience, and the ability to self-manage. From a systems and complexity perspective, health is best understood as a complex adaptive state that emerges from one's physical, social, emotional and cognitive experiences, i.e., health arises from the dynamics within a somato-psycho-socio-semiotic (SPSS) frame [61,62]. A powerful illustration of this concept comes from a patient who asked: "Doctor I know I am dying, but tell me why I am feeling so healthy." This highlights the crucial distinction that a person’s state of health does not necessarily correspond to the presence or absence of disease or infirmity.

Health Service Organisation

The running of a health service organisation is driven by its value propositions as well as its financial imperatives, both are tightly linked and thus ‘determine’ its leadership and management decisions. Significant systemic differences exist between health services run as a commercial or a ‘common good’ endeavour [32,72,73]. We firmly believe that health services are primarily a common good ‘enterprise’ and thus, their structure, resourcing and outcome measures should be designed for its resulting interdependencies.

Health system re-designers should be reminded that no one actually chooses to become a patient, and no one can predict, if and when he or she may become a patient. ‘Patient’ means ‘the vulnerable’, and as a matter of principle, no one, and in particular no health professional, should ever be tempted or forced to exploit the vulnerable [74]. This obviously has implications for how we view and deal with the interdependent needs and capacities of the most disadvantaged in our community who are invariably unable to ‘buy care’ within a commercial health system frame [34].

Health System Orientation

Health system orientation has significant impacts on the dynamics of care delivery. A primary focus is principally person-centred, whereas a specialist focus is generally (or by default) disease-centred. Of note, the main differences between the two orientations relate to greater protocol adherence to care processes without impacting disease-specific outcomes. Primary care achieves these same disease-specific outcomes despite having to manage greater complexity [47], while simultaneously achieving better overall patient experiences and health outcomes and doing so at a significantly lower cost [49,75].

Leadership

Health system leadership must embrace a systems and complexity-informed system-wide governance and accountability frame [34,76]. Leaders bring people together and maintain their focus on the system’s (i.e. organisation’s) shared purpose and its core values [67]. This entails that leaders provide and adapt, based on open and free-flowing feedback, the required information and resources that constrain the activities and behaviours within and across the system’s organisational layers. Only then can the system as-a-whole achieve the outcomes commensurate with its purpose [13,77]. Systemic leadership is a constant balancing act; how to best adjudicate the inevitably emerging competing demands of very different stakeholder interests [78,79].

How It Can Be Done: Four Value-Driven Examples

The Mayo Clinic and the NUKA Health System provide compelling examples that illustrate the successful translation of a values and complex-adaptive systems thinking-frame to the operational management of a healthcare organisation (Mayo Clinic) and the transformation from a highly dysfunctional to a high-functioning health service (NUKA). Systems thinking underpins the de-novo creation of a primary care system (The Eastern Deanery AIDS Relief Program (EDARP)), and is the driver of a community-oriented health system (Costa Rica). These examples from very different geographic and organisational scales all share a fundamental commitment to the notion of value as ‘what matters most to the patient’ [80] (Tab 3).

**Table 3:** Values-Based Approaches to Health Care Delivery and Health System Design

Case Studies	Purpose
Mayo Clinic [42]	PURPOSE: Inspiring hope and promoting health through integrated clinical practice, education and research.
	VALUES
	· Our institutional primary value: The needs of the patient come first.
	· Our core values: Respect, integrity, compassion, healing, teamwork, innovation, excellence and stewardship.
	· Our values-driven culture.
EDARP [43,44]	PURPOSE: To provide affordable quality patient centered health care services in a Christ like manner with competence and excellence.
	VALUES
	1. Compassion
	2. Professionalism
	3. Patient Focus
	4. Quality
	5. Integrity
	6. Leadership and development
	7. Teamwork
	8. Diversity
	PURPOSE: A Native Community that enjoys physical, mental, emotional and spiritual wellness.
	CORPORATE GOALS AND OBJECTIVES (= values)
NUKA [45]	Shared Responsibility
	Achieve excellence in customer-owner satisfaction.
	Increase community awareness of SCF’s services and programs.
	Increase the level of ownership of customer-owners.
	Commitment to Quality
	Improve work environments and employee development systems with an emphasis on Alaska Native employees.
	Continuously improve systems and processes.
	Increase the number of Alaska Native employees in all job categories.
	Improve the safety of customer-owners and employees across all settings.
	Family Wellness
	Reduce the rate of domestic violence, child abuse and neglect.
	Reduce the rate of and improve the management of cancer.
	Reduce the incidence of suicide.
	Reduce the rate of obesity.
	Reduce the rate of substance misuse.
	Reduce the rate of and improve the management of diabetes.
	Improve oral health.
	Reduce the rate of and improve the management of cardiovascular disease.
	Improve customer-owner overall health.
	Operational Excellence
	· Improve the management of expenses.
	· Improve utilization of information technology and data support systems and services.
	· Improve SCF systems for third party revenue generation and collections.
Costa Rica Health Ministry [46]	PURPOSE: Guarantee the protection and improvement of the physical, mental and social health of the population, through the exercise of health leadership.
	INSTITUTIONAL VALUES
	Leadership
	Improve the capacity to motivate and influence social actors and the population, achieving consensus, credibility and trust to achieve health objectives and goals.
	Transparency
	Ensure that information on all institutional processes and decisions is public and available and accessible to social actors and the population.
	Proactivity
	Initiative to anticipate events in pursuit of institutional objectives.
	Efficiency
	Ability to achieve the desired results with the minimum possible resources.
	Excellence
	Be better every day, go further, with the best performance and with a view to achieving the highest level of performance and productivity.
	Service orientation
	Identify user needs, provide optimal service and give a timely and quality response.

In short, the Mayo brothers built their organisation based on their core values - 'the needs of the patient come first' - and an organisational approach of a seamlessly streamlined care process to maximise effectiveness and efficiency in care delivery. Inevitably, the value of ‘the needs of the patient come first’ led to changes in the organisation’s ‘commercial’ arrangements, resulting in the creation of the Mayo Foundation, which dedicates its profits to ‘improving the clinics' ability to better meet their patients’ needs’ [81].

The Eastern Deanery AIDS Relief Program (EDARP), operating in the informal settlements of Nairobi, has been driven by a commitment to addressing patients' needs. Initially focused on ‘Caring for people dying from HIV/AIDS’, it quickly evolved as both patients and providers identified additional requirements. These included the co-treatment for tuberculosis, prevention of mother-to-child transmission of HIV, and educational and social support for neglected or orphaned children. By adhering to its core values, EDARP fostered ongoing collaboration with government agencies and local non-government organisations (NGOs), ultimately enabling the emergence of a primary healthcare system informed by community needs in one of Kenya's most underserved areas [82,83].

The NUKA Health System, owned and operated by the local indigenous community it serves, is also founded on core values defined by the community itself. The community prioritised ‘A Native Community that enjoys physical, mental, emotional and spiritual wellness’. Through community consultation, three key expectations emerged - having a shared relationship with their primary care provider, being treated with courtesy, respect, and cultural understanding, and having access to care when needed. The leadership sees its role in promptly adapting the organisation's services based on patient and community feedback, which has resulted in high satisfaction, marked improvement in preventive health care, reduction in hospitalisation, urgent and emergency care needs, and the rate of referrals to secondary and tertiary care services [84]. 

In 1941, the Costa Rica government decided to design a single-payer-based healthcare system on the premise of “[guaranteeing] the protection and improvement of the physical, mental and social health of the population, through the exercise of health leadership.” To achieve this vision, the system had to continuously adapt. In the 1990s, it transformed into a robust, primary care-led healthcare system founded on five pillars - the integration of public health with primary care, the deployment of multidisciplinary teams embedded within communities, geographic empanelment, measurement and quality improvement at all levels, and the integration of digital technologies across the system. These reforms significantly enhanced access to care, improved the quality of services, and produced better health outcomes [85].

Starting a whole-of-system value discourse

Achieving a values-based health system is challenging in an environment where values are contested [86]. However, as Larsson et al [86] suggested, the sustainability and quality of the health system depend on all stakeholders engaging in open discourse to establish a shared understanding of the system they aim to create. This discourse might be best guided by contemplating Peter Drucker’s insight that "doing things right" (perfect disease-focused management) does not equate to "doing the right thing" (addressing the patient’s priorities and abilities to manage health and life issues in his/her context) [87]. Similarly, William Osler’s wisdom prompts us to ask: "Is it more important to know what sort of disease a patient has (disease focus) or what sort of patient has a disease (person/illness focus)" [40].

From our perspective, the ‘value in healthcare’ is best understood in terms of achieving the right outcomes, namely, 'what matters to this patient' [23]. Centering the health system around 'the needs of the patient as its core value focus' can achieve a lasting effective, efficient and equitable care system, leading to better outcomes for patients. This principle is exemplified by institutions such as the Mayo Clinic, and at the large scale, the Costa Rican healthcare system [85].

To realise this vision, we must cultivate leadership that understands the complexities of this challenge. True value creation in health care requires a shift from hierarchical [88] to complex adaptive or generative leadership, a model that recognises the necessity of variability in system designs according to the constraints imposed by local context [34,67,89,90].

Conclusions

This paper applies systems thinking to explore the diverse and often contrasting values that shape health care, ranging from business efficiency to patient-centred care. Achieving health outcomes that matter to patients, the ultimate goal of health care, requires a shared understanding of the value that balances ‘value preferences’ shaped by business prerogatives of efficiency and profit with humanistic prerogatives of receiving effective person-centredness care. This paper aims to foster a broad, community-wide discourse that informs the development of locally adapted health systems. Case studies from the Mayo Clinic and the Costa Rican healthcare system illustrate how a commitment to shared values can drive effective, efficient and equitable care delivery.

However, there is no single simple ‘formula or universal blueprint' for building a value-based health system, nor is there ONE definite ‘right’ solution; success depends on systems thinking as the intellectual framework that enables systemic leadership ensuring that all stakeholders remain focused on the health system’s purpose: 'to deliver outcomes that really matter to patients'. This process is inherently context-sensitive, dynamic and emergent, requiring a fundamental shift in perspective - viewing healthcare not as an expense but as a long-term investment. Establishing and sustaining a values-driven health system is an ongoing process within a learning organisation, requiring continuous adaptation, collaboration, and refinement.
